# Analysis of the microbial community structure and characteristic flavor of traditional fermented bean paste

**DOI:** 10.1002/fsn3.4322

**Published:** 2024-08-21

**Authors:** Aiguo Luo, Yuyu Guo, Min Zhu, Bianfang Hu

**Affiliations:** ^1^ Department of Biological Science and Technology Jinzhong University Jinzhong China; ^2^ Shanxi Center of Technology Innovation for Compound Condiment Jinzhong University Jinzhong China; ^3^ Food Science and Engineering Shanxi Agricultural University Jinzhong China

**Keywords:** bean paste, correlation, microbial community, volatile flavor

## Abstract

Bean paste is a traditional Chinese fermented condiment, which is loved by consumers for its pleasant flavor and nutritional properties. However, the microbial communities and related flavor compounds in bean paste remain unclear. We studied the correlation between the core functional microorganisms and flavor compounds in bean paste samples at different fermentation stages. Bacterial and fungal communities changed significantly during fermentation. The results indicated that the dominant bacterial genera were *Staphylococcus*, *Lactococcus*, and *Bacillus*. The dominant fungi were *Aspergillus* and *Lichtheimia*. During the early and late stages of bean paste fermentation, alcohols, hydrocarbons, esters, and phenols were the main flavor compounds detected. The types and proportions of alcohols and esters in the bean pastes fermented for different durations were similar, whereas the relative contents of phenols and hydrocarbons were higher in the later stages of fermentation. Correlation analysis revealed that core functional microorganisms played a key role in the fermentation process, and the potential correlation between bacterial genera and flavors was stronger than that of fungal genera. *Staphylococcus* abundance was significantly correlated with the flavor levels of ethyl oleate, ç‐pinene, diallyl disulfide, á‐myrcene, D‐limonene, and tetradecanoic acid, ethyl ester. The abundance of *Aspergillus* and *Lichtheimia* was significantly correlated with the flavor levels of á‐myrcene, ç‐pinene, and DL‐3‐phenyllactic acid. This study explored the relationship between microorganisms and flavor compounds in bean paste to deepen our understanding of the mechanism of flavor formation for bean paste.

## Graphical abstract

The study identified that *Staphylococcus*, *Lactococcus*, and *Bacillus* are the dominant bacterial genera, while *Aspergillus* and *Lichtheimia* are the dominant fungal genera in fermented bean paste. Volatile flavor compounds such as alcohols, esters, and phenols were significantly influenced by the presence and abundance of these core microorganisms. There is a strong correlation between *Staphylococcus* abundance and the levels of ethyl oleate, α‐pinene, diallyl disulfide, α‐myrcene, D‐limonene, and tetradecanoic acid ethyl ester, which are key flavor compounds in bean paste.

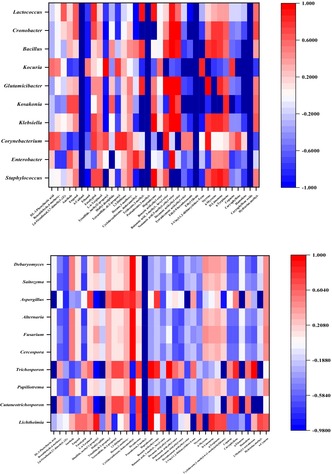

## 1 INTRODUCTION

Bean paste is a traditional Chinese fermented condiment popular among consumers because of its pleasant flavor and nutritious properties (Quang et al., 2017). Bean paste production employs a traditional open‐air, semisolid mixed fermentation process that consists of two main steps: making koji and fermenting sauce grains. Therefore, the production of traditional bean paste involves the interaction of multiple microorganisms and metabolites, with bacteria and fungi playing important roles in the fermentation process (Zhao et al., 2023). The primary functional fungi at the production stage are molds. They produce various enzymes by growing on raw materials, and these enzymes continue to break down the macromolecules in fava beans into small molecules of sugars, peptides, and amino acids. Proteins in the raw material are decomposed into various amino acids by proteases. In addition, amylase produced by *Aspergillus oryzae* converts starch in the raw material into sugars. Some of these sugars contribute to the flavor of the bean paste, some are converted into alcohol by yeast, and the rest are transformed into organic acids by various bacteria (Dong, 2016). Therefore, the microflora composition of bean paste significantly influences its flavor and quality. It is of great practical significance to identify the microorganisms present in bean paste and understand the changes in flavor substances. However, current studies on the correlation between microbial colonies and flavors in bean paste are not comprehensive.

High‐throughput sequencing technology and multi‐omics approaches have recently been widely used in various microbe‐related research fields. For example, Zhu et al. (2017) used polymerase chain reaction denaturing gradient gel electrophoresis (PCR‐DGGE) to analyze the predominant bacteria in different types of bean paste. Zhang et al. (2018) used traditional isolation methods and modern molecular biology techniques to analyze the microflora diversity. Cui et al. (2020) investigated the bacterial composition of bean paste by using high‐throughput sequencing. Regarding the study of volatile components of bean paste, Luo et al. (2018) used gas chromatography–mass spectrometry to determine the volatile flavors in the postfermentation period in Pixian bean paste samples and detected over 140 volatile aroma compounds falling into nine categories. Li et al. (2021) used simultaneous distillation‐extraction (SDE) in conjunction with the aroma activity value method to characterize the aroma of bean paste. Regarding the correlation between microorganisms and flavors in fermented products, Wu et al. (2023) analyzed the relationship between microflora and nonvolatile flavors in sourdough during different fermentation periods using high‐throughput sequencing and ultra‐high‐performance liquid chromatography–quadrupole time‐of‐flight mass spectrometry (UPLC‐TOF‐MS). Wang et al. (2023) used the Illumina MiSeq sequencing technology and an electronic nose to investigate the relationship between fungal colonies and flavor characteristics in low‐temperature Daqu. Therefore, a deeper understanding of the detailed information on the correlation between microbial colonies and flavors in bean paste using advanced sequencing and spectral mass spectrometry technologies is required.

In this study, we explored the key microorganisms involved in the fermentation stage of bean paste and investigated their flavor compounds in the final product. First, this was achieved by dynamically monitoring changes in microbial composition and flavor compounds. Secondly, the relationship between these key microbial communities and essential flavor compounds was elucidated using Pearson's correlation coefficient analysis to establish a theoretical framework for investigating the effect of microbial communities on the generation of flavor compounds during various fermentation stages.

## 2 MATERIALS AND METHODS

### 2.1 Sample preparation and collection

Moldy bean paste (Haitong Ende Foods Co., Ltd.) (500 g) and white wine (containing 45% ethanol, purchased from Beijing Erguotou Wine Co., Ltd.) (250 mL) were placed in a fermenter and left to ferment for 24 h at room temperature. Then, we added capsicum frutescens (Guoren Yi flagship store) (1 kg) and auxiliaries (Sichuan Shicui Food Co., Ltd.) (50 g) and fermented at 45°C for 30 days, stirred the bean paste every 2 days until the end of the fermentation. The bean paste was studied for 1, 4, 7, 10, and 15 days and labeled DBJ1d, DBJ4d, DBJ7d, DBJ10d, and DBJ15d, respectively. Amplification was unsuccessful because of the low fungal content in the first‐day sample. All samples were collected from the four corners of the fermenter, including the bottom, middle, and surface layers. The samples were bagged and frozen in liquid nitrogen. The samples were then returned to the laboratory for further analysis.

### 2.2 DNA extraction and sequencing of amplicons

Microbial DNA was extracted using the PowerSoil DNA Isolation Kit (MO BIO Laboratories). Each DNA sample was subjected to three parallel extraction processes to reduce errors caused by these operations. The combined DNA was then measured to determine the final DNA concentration using a NanoDrop 2000 ultraviolet–visible spectrophotometer (Thermo Scientific, Wilmington, USA). DNA was analyzed by 1% agarose gel electrophoresis and spectrophotometry (260/280 nm optical density ratio) (Ye et al., 2024). The DNA was extracted using specific primers 338F (5′‐ACTCCTACGGGGAGGCAGCA‐3′) and 806R (5′‐GGACTACHVGGGTWTCTAAT‐3′) to amplify the highly variable region V3–V4 of the bacterial 16S ribosomal RNA gene by a thermocycling PCR system (2720 PCR Amplifier, ABI, USA). The internal transcribed spacer (ITS 1) region of the fungus was amplified using the forward primer ITS1F (5′‐GGAAGTAAAAGTCGTAACAAGG‐3′) and reverse primer ITS1R (5′‐GCTGCGTTCTTCATCGATGC‐3′). Subsequently, the DNA was stored at −80°C for the next step. PCR amplification was carried out under the following conditions: (a) initial denaturation at 98°C for 2 min; (b) denaturation at 98°C for 15 s; (c) annealing at 55°C for 30 s; (d) extension at 72°C for 30 s; (e) repeating steps (b–e) for 20 cycles; (f) stabilization for 5 min; and (g) steps (a–f) was repeated 20 times for each sample. Each sample was subjected to three replicates of library amplification, and each library was subjected to three parallel PCR technical replicates. The PCR products from the same library were then combined. A gel imager was used to detect the presence of PCR amplification bands, followed by gel purification and quantification. The constructed library was sequenced using an Illumina MiSeq PE300 sequencing platform by Shanghai Pai Senno Biological Technology Co., Ltd.

Based on the overlapping relationship of paired‐end sequencing, the sequences from each sample overlapped and were assembled using FLASH V1.2.7 software, generating raw sequence data. The spliced raw data were filtered using Trimmomatic v0.33 software to obtain high‐quality sequence data. Finally, the UCHIME v4.2 software was used to identify and eliminate chimeric sequences to obtain valid data. Using the USEARCH software, the samples' operational taxonomic unit (OTU) numbers were obtained by clustering at 97% similarity. The sequences of the OTUs were compared with those in the Silva (for bacteria) and Unite (for fungi) taxonomic databases. Corresponding microbial species were annotated using a comparison threshold of 70% (Zhao et al., 2019).

### 2.3 Determination of volatile flavor compounds

Ground samples (10 g) were mixed with saturated brine (4 mL) before being transferred to a 35‐mL headspace flask. As an internal standard 1,2‐dichlorobenzene (concentration 500 μg/L in methanol) (10 μL) was added to each sample. The vials were equilibrated in a water bath at 55°C for 15 min and then extracted at a constant temperature of 55°C for 30 min. After extraction, the coated fibers were promptly inserted into the gas chromatography inlet and heated to 240°C for 5 min. Each sample was extracted in triplicate. The main volatile compounds were analyzed by headspace solid‐phase microextraction coupled with gas chromatography–mass spectrometry (HS‐SPME‐GC‐MS) (GCMS6800 gas chromatograph, Jiangsu Tianrui Instrument Co., Ltd.), and the volatile compounds were separated on a J&W DB‐5 ms column (30 m × 0.25 mm, 0.25 μm). Helium was used as a carrier gas at a flow rate of 1 mL/min. The oven temperature was initially set to 40°C, maintained for 5 min, then raised to 85°C at a rate of 6°C/min, and maintained for 1.5 min. Subsequently, it was increased to 148°C at a rate of 2.5°C/min and maintained for 1 min, before being further increased to 280°C at a rate of 20°C/min and maintained for 1 min (Xu et al., 2023). The instrument was set to operate at 70 eV in the electron ionization (EI) mode, with a scanning range from 25 to 450 amu. Volatile fractions were identified by comparing their mass spectra with the NIST 11S mass spectrometry database and their linear retention index (RI) values relative to C_6_–C_20_ n‐alkanes with those published. Semiquantification was approximated by comparing the peak areas to those of the internal standard, based on a calibration factor of 1, which was then converted to values in the original paste (ng/g paste).

### 2.4 Statistical analysis

All the tests were conducted in triplicate, and the statistics were expressed as mean ± standard deviation (SD). The data were analyzed using analysis of variance (ANOVA) in IBM (SPSS) Statistical Package for the Social Sciences Statistics 26, and statistical significance was set at *p* < .0. Principal component analysis (PCA) and two‐way orthogonal partial least squares discriminant analysis (O_2_PLS‐DA) were conducted on volatile flavors using SIMCA 14.1. Pearson's correlation was used to examine the relationship between microbial content and metabolites in various samples. The data were plotted using Origin 2022 software.

## 3 RESULTS AND ANALYSIS

### 3.1 α‐Diversity of microbial communities

**FIGURE 1:**
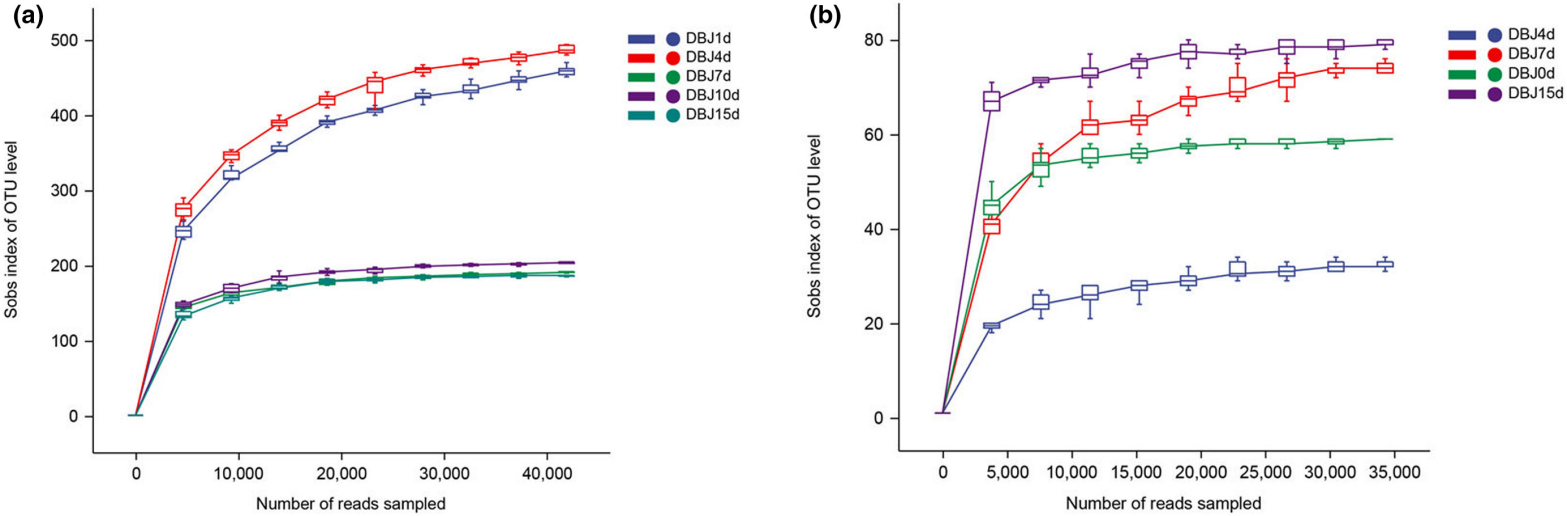
Rarefaction curve of bacteria (a) and fungi (b).

**FIGURE 2:**
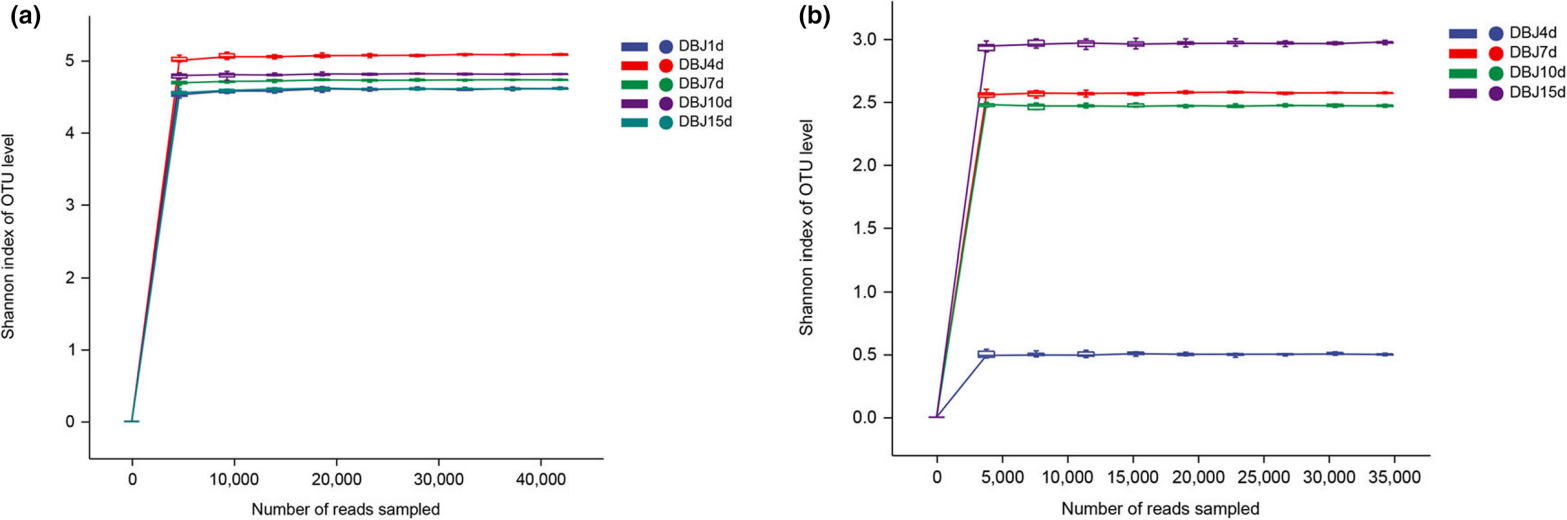
Shannon index curve of bacteria (a) and fungi (b).

Biodiversity can be used to characterize species richness in a sample. The index coverage was greater than 99% and accurately represented the microbial community. In general, the Sobs index represents the observed abundance, whereas the Shannon index is used to characterize the diversity of a community, which is constantly changing in terms of diversity and abundance. The sobs index exhibited an increasing, decreasing, and then increasing trend during bean paste fermentation (Figure 1a). This indicated that the bacterial community abundance in the bean paste samples was low on the first day, but with fermentation, the bacterial community abundance gradually increased until the fourth day. There was a decrease from the fourth to the fifteenth day, followed by increased bacterial community richness. This suggests that adding auxiliary materials may have inhibited the growth of exogenous bacteria. After fermentation, the bacterial community abundance recovered, indicating that the final bean paste product contained the highest number of bacteria. The Sobs indices of the fungi also increased, decreased, and then increased. They remained low and did not change significantly throughout the fermentation process (Figure 1b). This indicated that the abundance of bacteria in bean paste was higher than that of fungi. The Shannon's index results showed that the diversity of the bacterial communities remained stable (Figure 2a,b). On the seventh day, the diversity of the fungal community in bean paste changed significantly, suggesting an increase in fungal community diversity over time.

### 3.2 Venn diagram of OTU distribution

**FIGURE 3:**
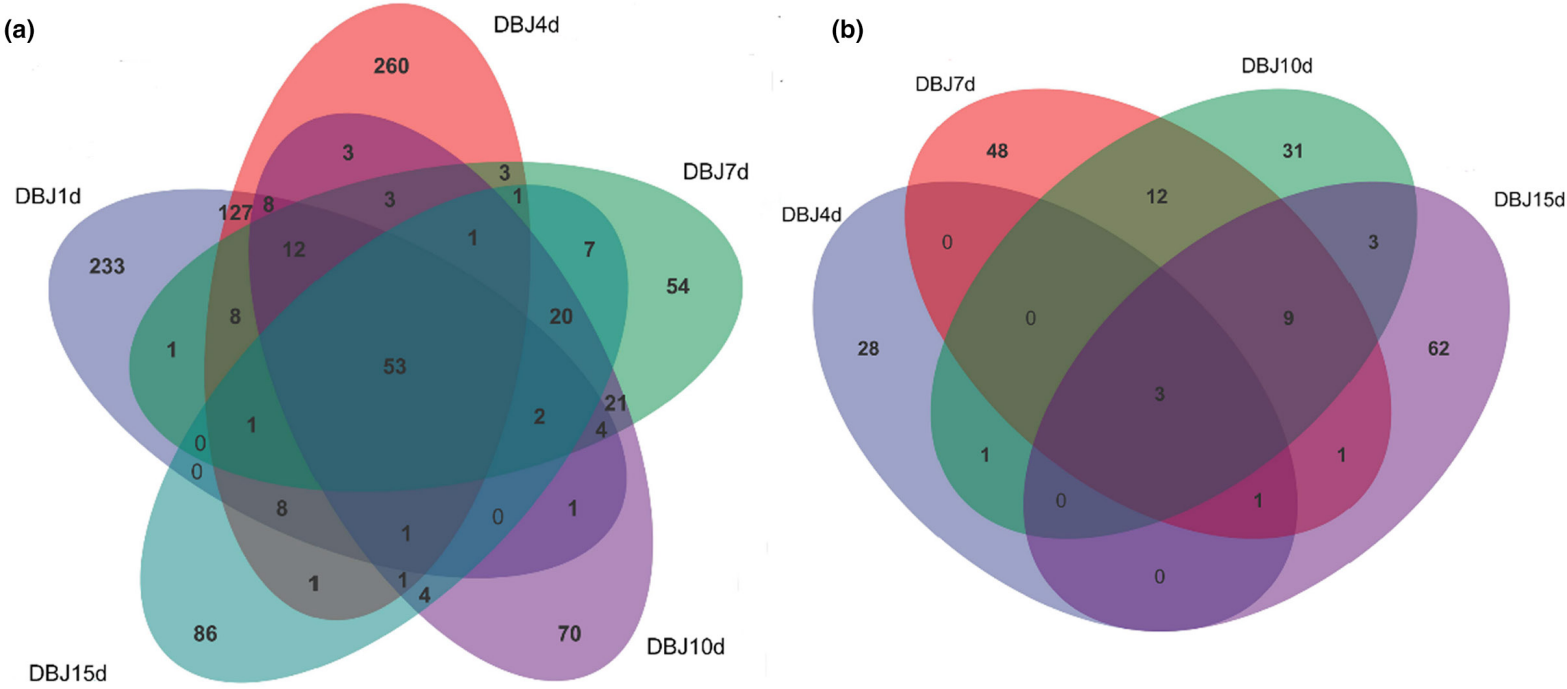
Venn diagram of OTU distribution of bacteria (a) and fungi (b).

The Venn diagram shows the number of OTUs unique to each sample and those shared among the samples. It also illustrates the similarities and overlaps in the composition of OTUs across the samples (Zhou et al., 2018). The number of OTUs of bacteria and fungi in DBJ1d, DBJ4d, DBJ7d, DBJ10d, and DBJ15d was 53 and 3, respectively, accounting for 7.01% and 1.74% of the total number of OTUs, respectively (Figure 3). The numbers of OTUs unique to the bacteria were 233, 260, 54, 70, and 86. The numbers of OTUs unique to fungi were 28, 48, 31, and 62. This suggests that differences in the microorganisms are present during bean paste fermentation. The number of species shared during the fermentation of bean paste was low as a proportion of the total number. This suggests that distinct species play crucial roles in shaping the flavor of bean paste.

### 3.3 Taxonomic composition and dynamic succession of microbial communities

**FIGURE 4:**
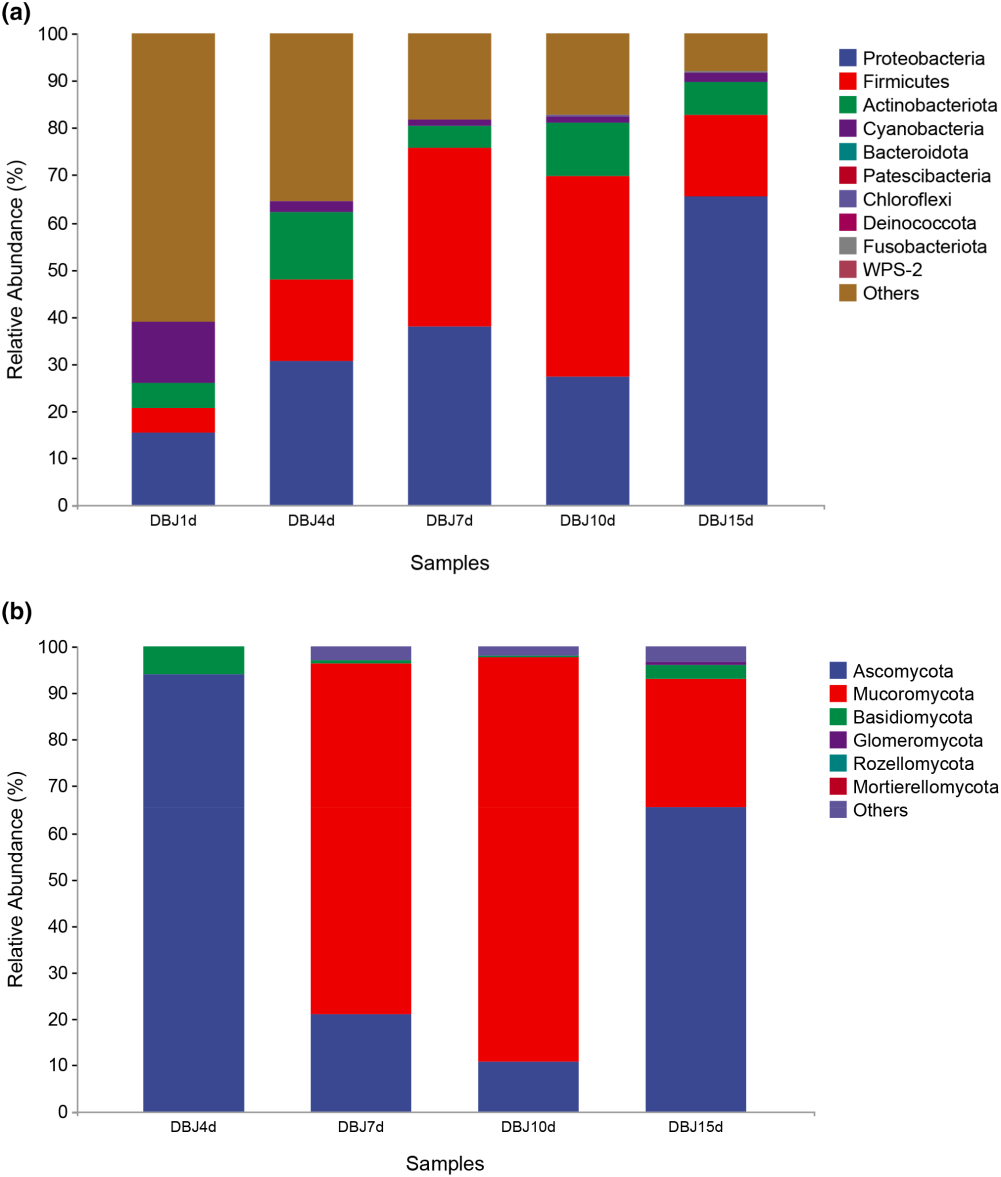
Histogram of relative abundance of bacteria (a) and fungi (b) at the phylum level.

**FIGURE 5:**
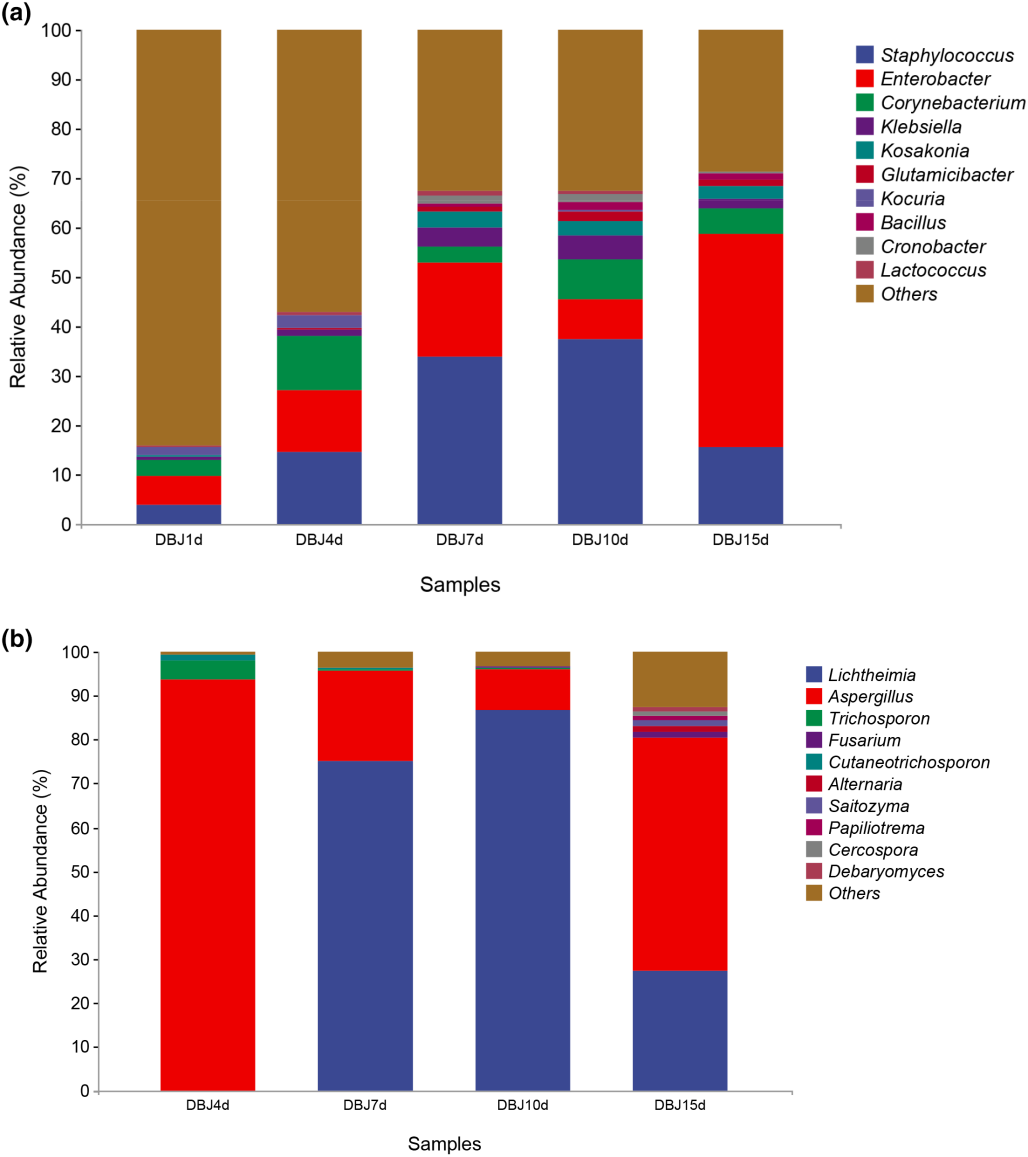
Histogram of relative abundance of bacteria (a) and fungi (b) at the genus level.

Figures 4 and 5 show detailed information about each taxon in the different phylogenies. At the phylum level, the bacteria in all the tested samples were predominantly Proteobacteria (65.33%), Firmicutes (17.58%), Actinobacteria (6.67%), and Cyanobacteria (2.19%). Microbial diversity at the fungal phylum level was relatively homogeneous, with the dominant phyla being Ascomycota (65.61%), Mucoromycota (27.26%), and Basidiomycota (3.15%). Proteobacteria and Firmicutes detected at the early stage of fermentation showed a significant increase (*p* < .05) as fermentation progressed and were the dominant bacterial phyla during the fermentation of bean paste.

At the genus level, the predominant genera in the bacterial community were *Lactococcus*, *Staphylococcus*, and *Bacillus*, representing 40.15% of the total bacterial population. Among these, *Staphylococcus* abundance increased and was dominant throughout the fermentation process. *Staphylococcus* can decompose glucose, maltose, and sucrose, which play important roles in the fermentation of bean paste (Yang, Deng, & Li, 2021; Yang, Niu, et al., 2021). *Bacillus* gradually reached a stable state during fermentation. In Korea, the primary source of *Lactococcus* is soy sauce. Its relative abundance increased and then decreased during the fermentation of bean paste and has remained relatively stable since then (Zhou et al., 2017). Microorganisms were present in the samples that significantly contributed to the quality of the bean paste. Most identified microorganisms could adapt to environmental changes and thrive under high salt, high sugar, and normal conditions. *Staphylococcus* and *Bacillus* spp. may affect volatile compounds in bean paste during fermentation (Li, 2020). Thus, the bacteria present in bean paste may modify the fermentation environment, resulting in a characteristic ester aroma and a fundamentally sour flavor profile.

### 3.4 Analysis of volatile flavor compounds in bean paste

HS‐SPME‐GC‐MS was used to analyze the volatile flavor compounds during bean paste fermentation. Seventy‐seven volatile compounds were identified, including one acid, two aldehydes, three phenols, five alcohols, seven sulfur‐containing compounds, nine alkanes, 12 esters, 23 olefins, and 15 other compounds (Table 1). The total volatile compound content increased gradually during fermentation. Alcohols and esters are the primary flavors found in bean paste (Zhao, 2021). Alcohols are products of microbial metabolism or the reduction of the carbonyl group in alcoholic fermentation. These precursors are crucial for long‐chain ester production. They also significantly affect the aroma and flavor of sauces and spirits (Guo et al., 2023). Esters have a positive effect on the aroma of bean paste. Examples include tetradecanoic acid ethyl ester, which has a coconut aroma and a sweet beeswax‐like flavor; hexadecanoic acid ethyl ester, which has a faint waxy and creamy aroma; and ethyl oleate, commonly used as a spice and cosmetic ingredient. Phenols, such as eugenol, are primarily characterized by their smoky and medicinal properties (Zhang et al., 2022). Olefins typically have a pleasant aroma and sweet flavor (Li et al., 2019). Elemene may be derived from ginger and diallyl disulfide may be derived from garlic volatilization (Zhao et al., 2021). In addition, acids are usually involved in esterification reactions to produce esters and significantly influence the flavor of bean paste (Ma et al., 2023). During the bean paste fermentation, various volatile compounds were detected that contributed to its characteristic flavor.

**TABLE 1:** Relative content of volatile flavor compounds in traditional bean paste.

Category	Name	Molecular formula	Relative content/%				
			1 day	4 days	7 days	10 days	15 days
**Acids**							
1	DL‐3‐Phenyllactic acid	C9H10O3	0.19 ± 0.02	0.71 ± 0.01	0.40 ± 0.01	0.27 ± 0.02	0.42 ± 0.01
**Aldehyde**							
1	Benzaldehyde, 4‐methoxy‐	C8H8O2	1.06 ± 0.01	1.28 ± 0.03	0.25 ± 0.02	1.54 ± 0.01	0.21 ± 0.01
2	2,6‐Octadienal, 3,7‐dimethyl‐,(Z)‐	C10H16O	0.56 ± 0.02	0.18 ± 0.02	–	0.36 ± 0.01	0.09 ± 0.01
**Phenols**							
1	Anethole	C10H12O	14.76 ± 1.2	8.57 ± 1.1	20.83 ± 2.30	12.55 ± 1.20	20.38 ± 3.10
2	Eugenol	C10H12O2	0.31 ± 0.01	0.24 ± 0.01	0.49 ± 0.01	0.31 ± 0.02	0.36 ± 0.02
3	à‐Cadinol	C15H26O	0.16 ± 0.01	0.18 ± 0.02	–	–	–
**Alcohols**							
1	Ethanol	C2H6O	5.04 ± 0.50	4.08 ± 0.40	–	3.10 ± 0.20	–
2	Eucalyptol	C10H18O	1.16 ± 0.03	1.26 ± 0.01	–	1.32 ± 0.02	1.11 ± 0.01
3	L‐à‐Terpineol	C10H18O	0.70 ± 0.02	1.15 ± 0.01	1.10 ± 0.01	–	1.03 ± 0.02
4	2‐Furanmethanol,5‐ethenyltetrahydro‐à,à,5‐trimethyl‐, cis	C10H18O2	–	–	0.15 ± 0.02	0.13 ± 0.01	0.13 ± 0.01
5	1,6,10‐Dodecatrien‐3‐ol,3,7,11‐trimethyl‐,(E)‐	C15H26O	0.16 ± 0.01	0.11 ± 0.01	1.58 ± 0.02	0.10 ± 0.01	0.08 ± 0.01
**Sulfur‐containing compounds**							
1	Dimethyl trisulfide	C2H6S3	–	0.19 ± 0.01	0.2 ± 0.013	0.20 ± 0.02	0.25 ± 0.01
2	Disulfide, methyl 1‐propenyl	C4H8S2	–	0.13 ± 0.01	–	0.19 ± 0.01	0.17 ± 0.01
3	Trisulfide, methyl 2‐propenyl	C4H8S3	–	4.15 ± 0.50	2.92 ± 0.05	2.75 ± 0.06	–
4	Diallyl sulfide	C6H10S	0.23 ± 0.02	0.50 ± 0.01	0.28 ± 0.01	0.21 ± 0.01	0.28 ± 0.02
5	Diallyl disulfide	C6H10S2	4.24 ± 0.30	4.37 ± 0.40	4.48 ± 0.50	4.32 ± 0.30	4.69 ± 0.20
6	Trisulfide, di‐2‐propenyl	C6H10S3	0.95 ± 0.03	2.86 ± 0.01	2.92 ± 0.02	2.38 ± 0.04	2.79 ± 0.02
7	Tetrasulfide, di‐2‐propenyl	C6H10S4	–	0.46 ± 0.02	0.08 ± 0.01	0.26 ± 0.01	0.31 ± 0.02
**Alkanes**							
1	1,3‐Dithiane	C4H8S2	0.24 ± 0.01	0.85 ± 0.01	0.51 ± 0.01	0.62 ± 0.02	0.62 ± 0.02
2	Hexane, 3‐methoxy‐	C7H16O	1.58 ± 0.05	4.15 ± 0.20	3.68 ± 0.02	–	3.91 ± 0.10
3	Cyclotetrasiloxane, octamethyl‐	C8H24O4Si4	–	–	0.33 ± 0.01	0.38 ± 0.02	0.41 ± 0.01
4	Cyclohexasiloxane, dodecamethyl‐	C12H36O6Si6	–	–	0.71 ± 0.01	0.75 ± 0.01	0.84 ± 0.02
5	Tridecane, 2‐methyl‐	C14H30	0.68 ± 0.01	0.87 ± 0.01	1.65 ± 0.02	1.59 ± 0.05	–
6	Hexadecane	C16H34	0.13 ± 0.02	–	0.31 ± 0.01	0.22 ± 0.01	0.24 ± 0.01
7	Pentadecane, 3‐methyl‐	C16H34	0.09 ± 0.01	–	–	–	0.40 ± 0.02
8	Pentadecane, 2‐methyl‐	C16H34	0.16 ± 0.01	0.14 ± 0.01	3.96 ± 0.30	0.37 ± 0.02	0.40 ± 0.02
9	Heptadecane	C17H36	0.13 ± 0.01	–	0.33 ± 0.01	–	0.16 ± 0.01
**Esters**							
1	Benzoic acid, ethyl ester	C9H10O2	0.75 ± 0.02	2.43 ± 0.05	1.35 ± 0.04	1.65 ± 0.01	1.25 ± 0.02
2	Butanoic acid, 3‐methyl‐, hexyl ester	C11H22O2	–	–	0.29 ± 0.01	3.26 ± 0.02	0.30 ± 0.01
3	Ethyl trans‐4‐decenoate	C12H22O2	–	0.21 ± 0.01	0.73 ± 0.01	1.30 ± 0.02	0.62 ± 0.01
4	Nonanoic acid, 5‐methyl‐,ethyl ester	C12H24O2	0.07 ± 0.01	0.12 ± 0.02	0.21 ± 0.01	–	–
5	Decanoic acid, ethyl ester	C12H24O2	0.06 ± 0.01	–	–	0.47 ± 0.01	0.19 ± 0.02
6	Dodecanoic acid, ethyl ester	C14H28O2	0.37 ± 0.02	0.37 ± 0.01	0.15 ± 0.01	0.21 ± 0.01	0.10 ± 0.01
7	Tetradecanoic acid, ethyl ester	C16H32O2	1.26 ± 0.04	1.54 ± 0.03	0.36 ± 0.01	0.60 ± 0.02	0.25 ± 0.01
8	1,6‐Octadien‐3‐ol,3,7‐dimethyl‐,2‐aminobenzoate	C17H23NO2	0.11 ± 0.01	0.09 ± 0.01	0.08 ± 0.01	–	–
9	Pentadecanoic acid, ethyl ester	C17H34O2	0.23 ± 0.02	0.26 ± 0.01	0.12 ± 0.02	0.28 ± 0.01	0.10 ± 0.01
10	Ethyl‐9‐hexadecenoate	C18H34O2	0.53 ± 0.01	0.67 ± 0.02	0.13 ± 0.01	0.26 ± 0.01	–
11	Hexadecanoic acid, ethyl ester	C18H36O2	1.63 ± 0.03	0.11 ± 0.01	0.60 ± 0.02	1.29 ± 0.01	0.51 ± 0.01
12	Ethyl Oleate	C20H38O2	1.60 ± 0.05	2.26 ± 0.20	–	–	–
**Olefin**							
1	1‐Propene, 1‐(methylthio)‐,(E)‐	C4H8S	0.13 ± 0.01	0.15 ± 0.01	0.08 ± 0.01	0.11 ± 0.01	–
2	3‐Vinyl‐1,2‐dithiacyclohex‐5‐ene	C6H8S2	0.83 ± 0.02	0.85 ± 0.02	0.55 ± 0.02	0.33 ± 0.01	0.53 ± 0.01
3	3‐Vinyl‐1,2‐dithiacyclohex‐4‐ene	C6H8S2	1.40 ± 0.04	0.99 ± 0.01	0.61 ± 0.02	0.50 ± 0.02	0.63 ± 0.02
4	Styrene	C8H8	–	–	0.18 ± 0.01	0.19 ± 0.02	0.18 ± 0.01
5	(1R)‐2,6,6‐Trimethylbicyclo[3.1.1]hept2‐ene	C10H16	0.15 ± 0.01	0.39 ± 0.01	–	0.61 ± 0.01	0.44 ± 0.02
6	à‐Pinene	C10H16	0.15 ± 0.01	0.14 ± 0.02	0.53 ± 0.01	0.61 ± 0.01	0.19 ± 0.02
7	Camphene	C10H16	0.50 ± 0.01	0.88 ± 0.02	1.34 ± 0.20	1.64 ± 0.30	1.20 ± 0.20
8	á‐Myrcene	C10H16	0.21 ± 0.02	0.37 ± 0.01	0.74 ± 0.01	0.85 ± 0.01	0.72 ± 0.02
9	D‐Limonene	C10H16	0.83 ± 0.01	2.17 ± 0.30	4.09 ± 0.50	4.28 ± 0.50	3.73 ± 0.30
10	ç‐Terpinene	C10H16	0.28 ± 0.01	0.38 ± 0.01	0.46 ± 0.02	0.42 ± 0.01	0.43 ± 0.02
11	Cyclohexene, 1‐methyl‐4‐(1‐methylethylidene)‐	C10H16	0.16 ± 0.01	0.13 ± 0.01	–	0.17 ± 0.01	0.16 ± 0.01
12	3‐Cyclohexen‐1‐ol,4‐methyl‐1‐(1‐methylethyl)‐,(R)‐	C10H18O	0.82 ± 0.01	0.76 ± 0.02	0.79 ± 0.01	0.67 ± 0.01	0.77 ± 0.02
13	3‐Cyclohexene‐1‐methanol,à,à,4‐trimethyl‐,acetate	C12H20O2	0.49 ± 0.01	0.50 ± 0.02	0.41 ± 0.01	0.27 ± 0.01	0.37 ± 0.01
14	Copaene	C15H24	0.99 ± 0.01	0.97 ± 0.01	0.72 ± 0.02	0.87 ± 0.01	0.72 ± 0.01
15	Caryophyllene	C15H24	2.14 ± 0.20	2.42 ± 0.30	1.88 ± 0.10	2.03 ± 0.02	1.83 ± 0.20
16	á‐Bisabolene	C15H24	1.17 ± 0.01	1.62 ± 0.02	1.58 ± 0.05	1.40 ± 0.01	1.55 ± 0.04
17	à‐Farnesene	C15H24	2.00 ± 0.02	2.28 ± 0.05	1.55 ± 0.02	1.72 ± 0.01	1.44 ± 0.01
18	ç‐Elemene	C15H24	0.13 ± 0.01	1.78 ± 0.02	0.18 ± 0.02	0.68 ± 0.01	0.62 ± 0.01
19	Bicyclo[3.1.1]hept‐2‐ene,2,6‐dimethyl‐6‐(4‐methyl‐3‐pentenyl)‐	C15H24	1.63 ± 0.01	0.46 ± 0.01	0.66 ± 0.02	0.18 ± 0.01	–
20	Humulene	C15H24	0.25 ± 0.01	0.25 ± 0.01	0.17 ± 0.01	0.15 ± 0.01	0.16 ± 0.01
21	Bicyclo[7.2.0]undec‐4‐ene,4,11,11‐trimethyl‐8‐methylene‐, [1R‐(1R*,4Z,9S*)]‐	C15H24	0.76 ± 0.01	0.14 ± 0.02	0.78 ± 0.01	0.80 ± 0.03	–
22	Caryophyllene oxide	C15H24O	0.19 ± 0.01	0.11 ± 0.01	–	–	–
23	2‐Methyl‐1‐tetradecene	C15H30	–	–	0.64 ± 0.01	0.57 ± 0.02	0.57 ± 0.01
**Other**							
1	Hydrazine, methyl‐	CH6N2	1.52 ± 0.01	0.77 ± 0.02	1.69 ± 0.03	1.61 ± 0.05	1.27 ± 0.03
2	Estragole	C10H12O	3.47 ± 0.20	3.26 ± 0.30	2.82 ± 0.10	3.26 ± 0.20	3.00 ± 0.20
3	o‐Cymene	C10H14	0.10 ± 0.01	0.19 ± 0.01	0.23 ± 0.01	–	0.23 ± 0.02
4	à‐Phellandrene	C10H16	0.06 ± 0.01	0.11 ± 0.01	–	0.10 ± 0.01	–
5	á‐Ocimene	C10H16	0.08 ± 0.01	0.13 ± 0.01	0.39 ± 0.02	0.22 ± 0.01	0.32 ± 0.02
6	1‐(3‐Methyl‐2‐butenoxy)‐4‐(1‐propenyl)benzene	C14H18O	–	1.14 ± 0.02	–	0.33 ± 0.01	0.24 ± 0.01
7	Cyclohexane,1‐ethenyl‐1‐methyl‐2,4‐bis(1‐methylethenyl)‐,[1S‐(1à,2á,4á)]‐	C15H24	0.55 ± 0.01	0.45 ± 0.02	–	0.53 ± 0.02	0.44 ± 0.01
8	1H‐Benzocycloheptene, 2,4a,5,6,7,8‐hexahydro‐3,5,5,9‐tetramet hyl‐, (R)‐	C15H24	0.79 ± 0.01	0.06 ± 0.01	–	1.51 ± 0.03	0.14 ± 0.01
9	Benzene,1‐(1,5‐dimethyl‐4‐hexenyl)‐4‐methyl‐	C15H22	3.14 ± 0.02	4.25 ± 0.20	3.96 ± 0.30	3.67 ± 0.02	3.76 ± 0.20
10	Naphthalene, decahydro‐4a‐methyl‐1‐methylene‐7‐(1‐methylethenyl)‐, [4aR‐(4aà,7à,8aá)]‐	C15H24	0.21 ± 0.01	–	–	0.28 ± 0.02	0.29 ± 0.01
11	Naphthalene,2,3,4,4a,5,6‐hexahydro‐1,4a‐dimethyl‐7‐(1‐methylethyl)	C15H24	0.21 ± 0.01	0.24 ± 0.02	0.31 ± 0.01	0.28 ± 0.02	0.29 ± 0.01
12	1,3‐Cyclohexadiene,5‐(1,5‐dimethyl‐4‐hexenyl)‐2‐methyl‐,[S‐(R*,S*)]	C15H24	5.40 ± 0.50	6.09 ± 0.30	7.02 ± 0.50	6.84 ± 0.40	6.75 ± 0.30
13	Naphthalene,1,2,4a,5,8,8a‐hexahydro‐4,7‐dimethyl‐1‐(1‐methylethyl)‐,[1S‐(1à,4aá,8aà)]‐	C15H24	0.53 ± 0.02	–	0.58 ± 0.02	0.54 ± 0.01	0.53 ± 0.01
14	Cyclohexene, 3‐(1,5‐dimethyl‐4‐hexenyl)‐6‐methylene‐,[S‐(R*,S*)]‐	C15H24	3.02 ± 0.01	3.73 ± 0.02	3.43 ± 0.02	3.40 ± 0.01	3.33 ± 0.01
15	1H‐Benzocycloheptene,2,4a,5,6,7,8‐hexahydro‐3,5,5,9‐tetramethyl‐(R)‐	C15H24	0.08 ± 0.01	–	1.58 ± 0.02	1.51 ± 0.01	1.47 ± 0.01

### 3.5 Correlation between microorganisms and key flavor compounds

**FIGURE 6:**
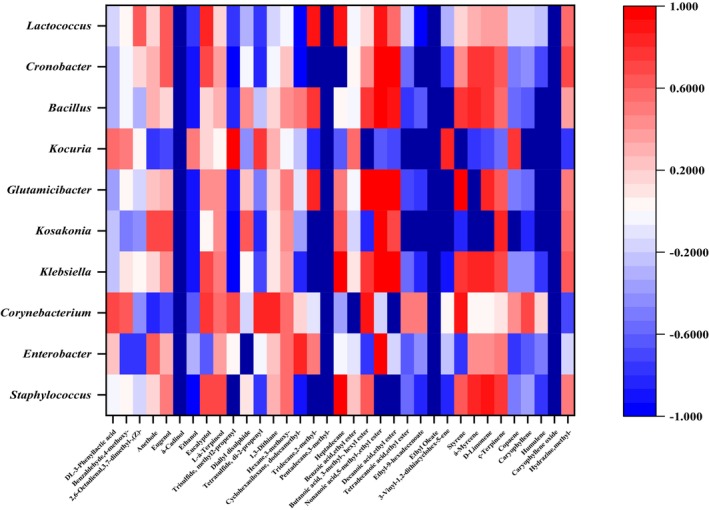
Heat map of correlation between bacterial communities and volatile flavor compounds in traditional bean paste.

**FIGURE 7:**
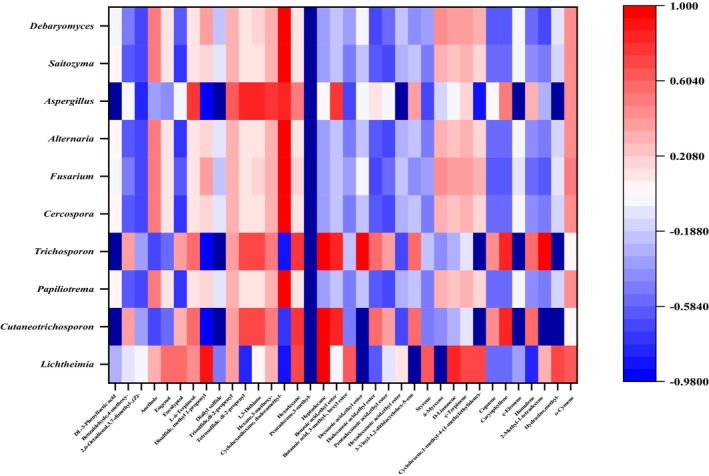
Heat map of correlation between fungal communities and volatile flavor compounds in traditional bean paste.

Studying the potential relationships between microbial communities and flavor compounds provides a framework for screening functional microorganisms. Resolving the relationship between microorganisms and flavor compounds is beneficial for exploring the potential flora responsible for flavor and controlling the flavor properties of fermented foods (Li et al., 2019). Substances that undergo significant changes in their volatile components during fermentation were correlated with core microorganisms to create heat maps (Yi et al., 2021). Pearson correlation coefficients were used to investigate the association between significantly different volatile flavor substances and core microorganisms (Figures 6 and 7).

*Staphylococcus* abundance significantly and positively correlated with the flavor levels of à‐Cadinol, ethanol, most alkanes, decanoic acid, ethyl ester, ethyl oleate, and ç‐Terpinene. The abundance of *Lactococcus* and *Bacillus* was significantly and positively correlated with the flavor levels of à‐Cadinol, pentadecane, 3‐methyl‐, and ethyl oleate (Figure 6). This suggests that multiple genera may be associated with a single flavor compound. *Lichtheimia* abundance was significantly and positively correlated with the flavor levels of pentadecane, 3‐methyl‐, decanoic acid, ethyl ester, á‐myrcene, and ç‐terpinene. *Aspergillus* abundance was significantly and positively correlated with the flavor levels of the acids, pentadecane, 3‐methyl‐, and ç‐elemene (Figure 7). These substances are primarily fruity, floral, and wine aromatic, which are crucial for the flavor of bean paste (Yang, Deng, & Li, 2021; Yang, Niu, et al., 2021).

## 4 CONCLUSION

Analyzing the relationship between microorganisms and flavor compounds is essential for exploring the potential functional flora responsible for flavor and controlling the flavor characteristics of fermented foods. In this study, we investigated the variation in microbial diversity and volatile flavor compounds in bean paste during fermentation. We analyzed the microbial community and volatile flavor compounds using high‐throughput screening and HS‐SPME‐GC‐MS. Additionally, we established a correlation between volatile flavors and dominant microorganisms based on the Pearson correlation coefficient. Two significantly different fungal genera and three significantly different bacterial genera were identified as being dominant. Of the 77 volatile flavor substances identified, 35 were significantly different. *Staphylococcus* abundance was highly correlated with the flavor levels of ethyl oleate, ç‐pinene, diallyl disulfide, á‐myrcene, and D‐limonene, which are characteristic flavor substances. The abundances of *Aspergillus* and *Lichtheimia* were significantly positively correlated with the flavor levels of á‐myrcene, ç‐pinene, and DL‐3‐phenyllactic acid. This study aimed to analyze the mechanism of flavor formation by the microbiota structure of bean paste, develop high‐quality bean paste production technology based on microbial regulation, and enhance the flavor and quality stability of the product.

## AUTHOR CONTRIBUTIONS

**Aiguo Luo:** Conceptualization (equal); funding acquisition (lead); investigation (equal); methodology (equal); resources (equal); writing – review and editing (equal). **Yuyu Guo:** Conceptualization (equal); formal analysis (equal); methodology (equal); visualization (equal); writing – original draft (equal). **Min Zhu:** Formal analysis (equal); writing – review and editing (equal). **Bianfang Hu:** Conceptualization (equal); funding acquisition (equal); project administration (equal); writing – review and editing (equal).

## CONFLICT OF INTEREST STATEMENT

The authors declare that they have no conflict of interest.

## Data Availability

The data that support the findings of this study are available from the corresponding author [Aiguo Luo] upon reasonable request.
